# Synthesis, Identification and Anti-Cancer Activity of 1-(4-Methylpent-2-enyl)-2-(4-phenylbut-2-enyl)disulfane

**DOI:** 10.3390/molecules15085671

**Published:** 2010-08-16

**Authors:** Chunxiao Ji, Fenglian Ren, Ming Xu

**Affiliations:** 1 College of Chemistry and Chemical Engineering, Central South University, Changsha, Hunan 410083, China; E-Mail: cx312@163.com (C.J.); 2 Research Institute for Molecular Pharmacology and Therapeutics, Central South University, Changsha, Hunan 410083, China

**Keywords:** 1-(4-methylpent-2-enyl)-2-(4-phenylbut-2-enyl)disulfane, synthesis, anti-cancer activity, XIAP

## Abstract

In this study, we synthesized 1-(4-methylpent-2-enyl)-2-(4-phenylbut-2- enyl)disulfane using sodium sulfide, 1-bromine-4-methyl-2-amylene and 1-(4-bromine-2- butylene)benzene as raw materials. The yield rate of target product was 84%. The structure of the target product was confirmed by GC-MS, ^1^H-NMR and elemental analysis. The results of anti-cancer activity experiments showed that 1-(4-methylpent-2-enyl)-2-(4- phenylbut-2-enyl)disulfane could significantly inhibit the proliferation, induce the apoptosis of CNE2 cells in a dose dependent manner, and could significantly enhance the activity of XIAP.

## 1. Introduction

Allicin is a natural allyl sulfide and one of the various sulfur compounds extracted from garlic, along with diallyl trisulfides, diallyl disulfides and so on [1,2,3,4,5]. Since 1980, allicin has attracted more and more attention because of its potential cancer prevention and anti-cancer effects [6]. At present, studies have shown that DADS (diallyl disulfide), a possible precursor of allicin, is a drug with broad-spectrum anti-cancer effects. It can inhibit the growth of various tumor cells, such as human colon cancer cells (HCT-15), human skin cancer cells (SK MEL-2), human gastric cancer cells, human breast cancer cells (MCF-7, KPL-1), and so on [7,8,9,10,11,12]. Certainly, study on the bioactivity of other sulfur compounds in this area continues. 

**Figure 1:**
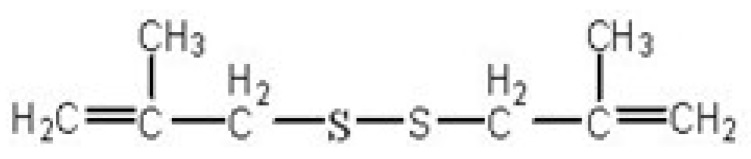
Structure of 1,2-bis(methylallyl)disulfane.

NPC (nasopharyngeal carcinoma) is a kind of malignant tumor with high incidence in the South- east Asia region. In the clinic, NPC therapy is usually based on radiation treatments, but the therapeutic effects are not satisfactory. In recent years, a lot of researchers have focused on investigating the anti-cancer mechanisms of active ingredients extracted from natural plants to prevent and treat cancer [13,14,15]. In this context, however, allicin, the main active ingredient of garlic, is unstable, and decomposes very easily during the extraction process. 1,2-bis(2-Methylallyl)disulfane (Figure 1) is a sulfur compound related to allicin with various biological activities [16,17,18]. Our previous work has demonstrated that 1,2-bis(2-methylallyl)disulfane could significantly inhibit the proliferation, and induce the apoptosis of human HepG2 cells. In this paper, we have successfully synthesized 1-(4-methylpent-2-enyl)-2-(4-phenylbut-2-enyl)disulfane using sodium sulfide, 1-bromine-4-methyl-2- amylene and 1-(4-bromine-2- butylene)benzene as raw materials, connecting isopropyl and benzyl groups to both ends of 1,2-bis(methylallyl)disulfane, and proved that the resulting 1-(4-methylpent-2-enyl)-2-(4-phenylbut-2-enyl)disulfane could significantly inhibit the proliferation and induce apoptosis. 

## 2. Results and Discussion

**Scheme 1:**
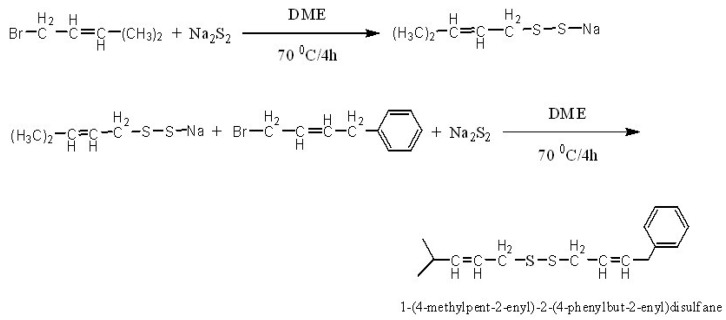
Synthesis route to 1-(4-methylpent-2-enyl)-2-(4-phenyl- but-2-enyl)-disulfane.

The synthetic route used is shown in Scheme 1. The target 1-(4-methylpent-2-enyl)-2-(4-phenyl- but-2-enyl)-disulfane was obtained as yellow oily substance in 84.0% yield and purity above 99%. 

In order to investigate the apoptosis effects on the CNE2 nasopharyngeal cancer cell line induced by 1-(4-methylpent-2-enyl)-2-(4-phenylbut-2-enyl)disulfane and its molecular mechanisms, the growth inhibition and apoptosis of CNE2 cells and the protein levels of XIAP were examined by MTT assay, flow cytometry and Western blot, respectively.

**Figure 2:**
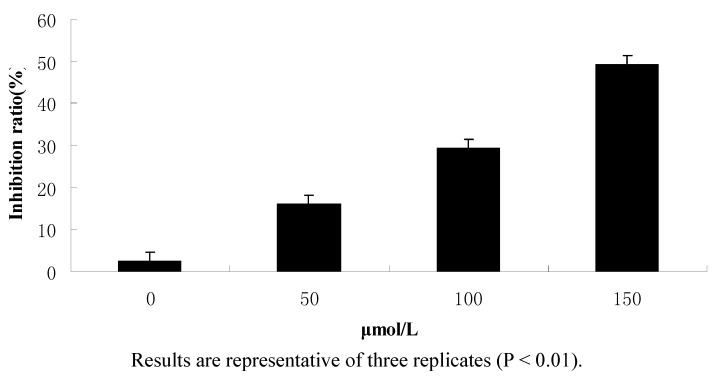
Results of the MTT assay.

To test the effect of 1-(4-methylpent-2-enyl)-2-(4-phenylbut-2-enyl)disulfane on CNE2 cells, cell viability was determined by the MTT assay. As shown in Figure 2, the CNE2 cells were exposed to drug concentrations of 0, 50, 100 and 150 μmol/L for 24 h, and the results showed that the inhibition ratios of treated with 50 μmol/L, 100 μmol/L and 150 μmol/L 1-(4-methylpent-2-enyl)-2-(4-phenyl- but-2-enyl)disulfane were 16.03%, 29.51% and 49.47%, respectively and the inhibition was evidently dose dependent. 

**Figure 3:**
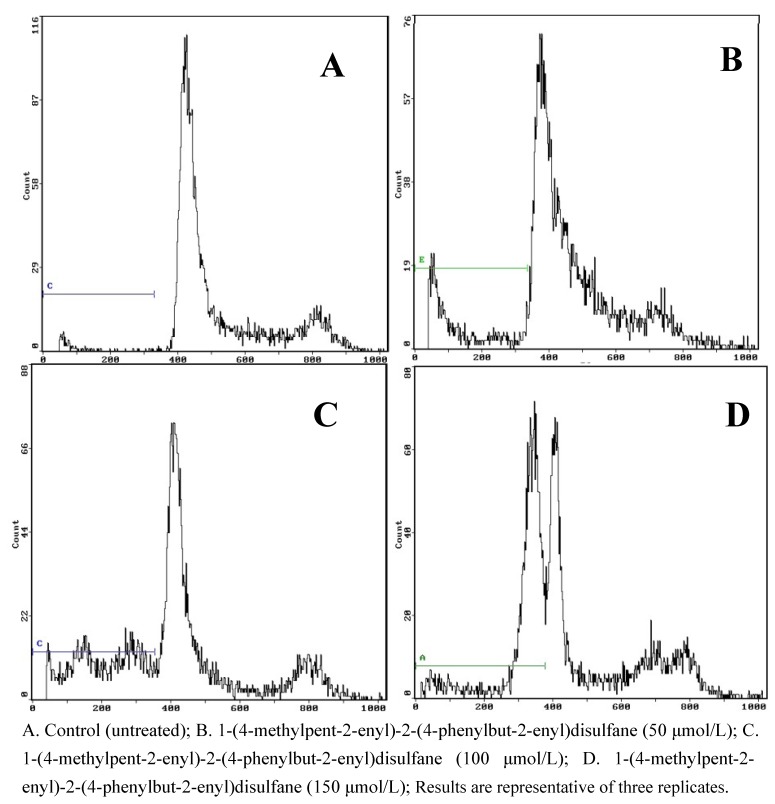
Effects of each group on apoptosis in in CNE2 cells.

**Figure 4:**
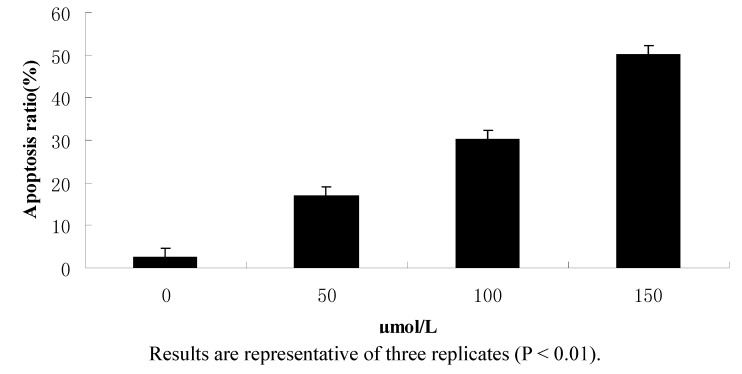
Results of the flow cytometry analysis.

To further examine the effects of 1-(4-methylpent-2-enyl)-2-(4-phenylbut-2-enyl) disulfane on apoptosis, flow cytometry was used to quantify the apoptotic state (Figure 3 and Figure 4). 

After treatment and incubation for 24 hr, the apoptosis ratios of cells treated with 50, 100 and 150 μmol/L 1-(4-methylpent-2-enyl)-2-(4-phenylbut-2-enyl) disulfane were 16.97%, 30.27% and 50.32%, respectively. The results also supported the notion that 1-(4-methylpent-2-enyl)-2-(4-phenylbut- 2-enyl)disulfane induced apoptosis of CNE2 cells in a concentration-dependent manner.

**Figure 5:**
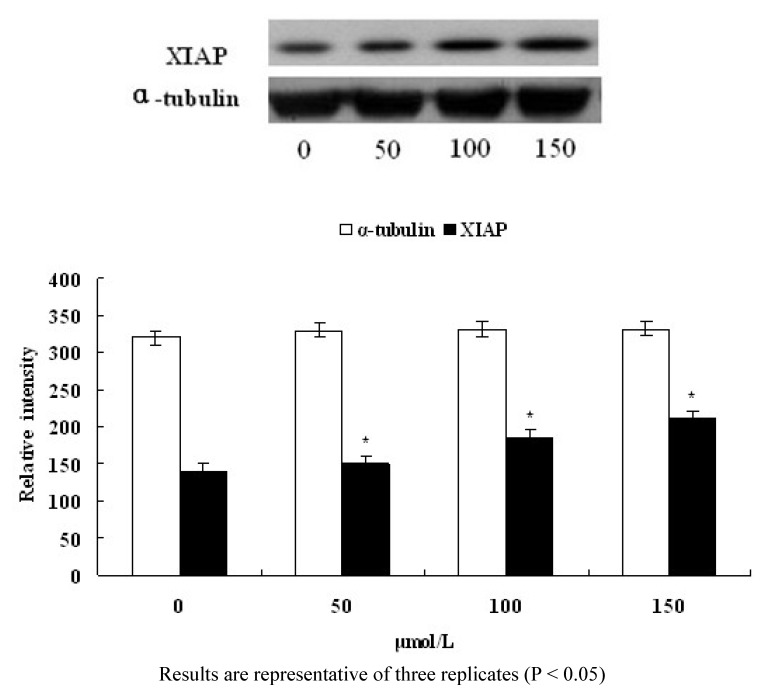
Effects of 1-(4-methylpent-2-enyl)-2-(4-phenylbut-2-enyl)disulfane on the protein expressions by Western blot.

XIAP (X-linked inhibitor of apoptosis protein) is an important member of the IAPS (inhibitor of apoptosis protein) family. To test the effect on XIAP when 1-(4-methylpent-2-enyl)-2-(4-phenylbut-2- enyl)-disulfane induced apoptosis of human CNE2 cells, we examined XIAP protein expression by Western blot analysis. After treatment with 1-(4-methylpent-2-enyl)-2-(4-phenylbut-2-enyl)disulfane (50, 100, 150 μmol/L, respectively) for 24 h, the expression of XIAP was significantly increased. When the concentration of 1-(4-methylpent-2-enyl)-2-(4-phenylbut-2-enyl)disulfane was increased, the expression protein of XIAP was also increased gradually (Figure 5), and our results showed that the activity of XIAP was enhanced significantly by the treatment. 

## 3. Experimental

### 3.1. General

3-Chloro-2-methylpropylene, 1-bromo-4-methyl-2-amylene, 1-(4-bromine-2-butylene)benzene and 1,2-dimethoxyethane (DME) were purchased from Fluka. Ether, benzophenone, sulphur, chloroform and carbinol were purchaed from Nanjing Chemical Reagents Co. 3-(4,5-Dimethylthiazol-2-yl)-2,5-diphenyltetrazolium bromide (MTT) and propidium iodide (PI) were purchased from Sigma. XIAP and α-tubulin were purchased from Cell Signaling. DME was dried with metal sodium using benzophenone as the indicator before being used.^ 1^H-NMR was recorded on a Bruker DRX-500 spectrometer at 298 K, elementary analysis was performed on a Perkin-Elmer 240 C analytic instrument. GC-MS analyses were performed on a HP6890 gas chromatograph equipped with a HP 5973 mass selective detector (MS) and a fused-silica-capillary column, DB-5, (30 m × 0.32 mm).

### 3.2. Synthesis of 1-(4-methylpent-2-enyl)-2-(4-phenylbut-2-enyl)disulfane

Sodium metal sheet (12.7g, 0.55 mol) was added to DME (100 mL) with fast stirring, and then sulphur powder (16.0g, 0.50 mol) was added after the sodium metal was completely dispersed, and stirring of the mixture was continued at the room temperature for 2 h, to give the product Na_2_S_2 _, which was sealed and kept in a cool place [19,20,21]. Na_2_S_2 _(50 mL) was transferred into a round bottom flask (250 mL), then DME (50 mL) which contained 1-bromo-4-methyl-2-amylene (40.8 g, 0.25 mol) was added dropwise into the round bottom flask with continuous stirring, and then reacted in a 70 °C oil bath for 4 h. After that, solvent was removed by the rotary evaporator under vacuum, and a bright yellow oily matter was thus obtained. The oily matter was added into distilled water (50 mL) and dispersed with ultrasound, then extracted with ether (20 mL) five times, and extracted with chloroform three times. The combined extracts was evaporated under vacuum to remove the solvent. The product was finally purified by silica gel column chromatography, using chloroform/methanol (v/v = 99/1) as mobile phase [22]. 

### 3.3. Characterization of 1-(4-methylpent-2-enyl)-2-(4-phenylbut-2-enyl)disulfane

^1^H-NMR: (CDCl_3_) δ (ppm): 7.04-7.02 (2H, d, 3,5-Ph-H), 6.91-6.81 (3H, t, 2,4,6-Ph-H), 6.79-6.79 (1H, t, benzyl-CH=), 5.56-5.47 (3H, t, -CH=CH- + benzyl-CH= CH-), 3.19-3.16 (6H, t, -CH_2_-), 2.22-2.19 (1H,d, (CH3)_2_CH-), 1.33 (6H, s, -CH_3_) ppm; Elemental analysis: found (calcd.) (%): C, 68.89 (69.01); H, 7.92 (7.96); S, 23.14 (23.03); GC-MS analysis (*m/z*): 278 (M^+^), 163, 147, 138, 131, 115, 109, 104, 99, 95, 94, 91, 83, 72, 69, 55, 44, 40.

### 3.4. Cell culture

CNE2, a human nasopharynaeal carcinoma cell line, were cultured in RPMI1640 with 10% heat-inactivated fetal bovine serum (FBS), benzylpenicillin (100 kU/L) and streptomycin (100 mg/L) at 37 °C in an incubator containing humidified air with 5% CO_2_.

### 3.5. Cell viability assay

To assess the cytotoxic effects of DADS in CNE2 cells, we used a3-(4,5-dimethylthiazol-2-yl)-2,5- diphenyltetrazolium bromide (MTT) tetrazolium salt reduction assay [23]. In this assay, the MTT is used as a colorimetric substrate for measuring cell viability. When cells are injured, there is an alteration in the cellular redox activity such that cells are unable to reduce the dye [7]. Cells were seeded into 96-well plates at 1 × 10^4 ^cells per well 24 h before treatment. The cultures were then rinsed in phenol-free RPMI1640 medium and incubated with respective test substances in phenol-free and serum-free RPMI1640 for 24 h. In the inhibition test, cells were treated with 1-(4-methylpent-2-enyl)-2-(4-phenylbut-2-enyl) disulfane. At the end of this time interval, 20 μL (5 mg/mL) MTT was added to each well, and after incubation at 37 °C for 4 h, the MTT solution was removed and 200 μL of dimethylsulfoxide (DMSO) was added to dissolve the crystals. The absorbance of each well was measured at 570 nm.

### 3.6. Flow cytometry analysis

Cells were seeded into 100 mL cell culture bottles at 12 × 10^6^ cells 24 h before treatment. Then cells were treated according to the aforementioned method and incubated for 24 h. Afterwards, floating and adherent cells were collected, washed three times with PBS (pH 7.4) and fixed for 24 h with cool alcohol at 4 °C. 1 mL cell suspension (10^6^/mL) was washed ken for three times with cooled PBS, treated with RNase for 30 min at 37 °C, stained it with PI for 30 min at 37 °C in a dark environment, and taken for flow cytometry analysis.

### 3.7. Western blotting

The cells were taken in the logarithmic growth phase, treated according to the aforementioned method and incubated for 24 h. After fragmentation on ice for 20 min, the lysates were centrifuged at 15,000 ×g for 10 min at 4 ºC, the protein was collected, quantitated with the BCA method, electrophoresed and isolated by the SDS-PAGE (10%) using the electrotransfer method, blocked and hybridized on the cellulose nitrate film. Then the protein expression of cells was detected using the ECL Western blotting method. The densities of protein bands were calculated using the Quantity One software.

### 3.8. Statistics

Data are expressed as mean ± S.D of three independent experiments and were evaluated by one-way analysis of variance (ANOVA). Significant differences were established at P < 0.05.

## 4. Conclusion

The target product 1-(4-methylpent-2-enyl)-2-(4-phenylbut-2-enyl)disulfane was characterized by GC-MS, ^1^H-NMR and elemental analysis. The results of anti-cancer activity experiment showed that this compound could significantly inhibit the proliferation of CNE2 cells, induce apoptosis in a dose dependent manner, and could significantly enhance the activity of XIAP.
